# HIV-1 Treatment-as-Prevention: A Cohort Study Analysis of Serodiscordant Couples in Rural Southwest China

**DOI:** 10.1097/MD.0000000000000902

**Published:** 2015-06-19

**Authors:** Zhenzhu Tang, Guanghua Lan, Ying Qing Chen, Qiuying Zhu, Xiaoyi Yang, Zhiyong Shen, Yi Chen, Heng Zhang, Wei Kan, Hui Xing, Yuhua Ruan, Yiming Shao

**Affiliations:** From the Guangxi Center for Disease Control and Prevention, Nanning, China (ZT, GL, QZ, ZS, YC); Fred Hutchinson Cancer Research Center and University of Washington, Seattle, WA, USA (YC); School of Public Health, Guangxi Medical University, Nanning (XY); State Key Laboratory for Infectious Disease Prevention and Control (SKLID), Beijing (HZ, WK, HX, YR, YS); and Collaborative Innovation Center for Diagnosis and Treatment of Infectious Diseases, Hangzhou, China (HZ, WK, HX, YR, YS).

## Abstract

The Chinese national observational cohort study suggests that the treatment-as-prevention (TasP) approach can be an effective public health HIV-1 prevention strategy. However, results from that study may have been biased because the follow-up time of index patients prior to their initiation of antiretroviral therapy (ART) was excluded. In this study, we correct for such bias by using an extended time-dependent Cox regression model to conduct a cohort study analysis of serodiscordant couples in Guangxi of China, inclusive of all follow-up time.

During the follow-up of this observational cohort study of HIV-1 sero-discordant couples, the positive index partners may have never be treated with ART, or enter untreated but subsequently began treatment, or may have been treated immediately upon entry into the public health system. The treatment effectiveness of ART in HIV-1 acquisition among HIV-negative partners is assessed by the extended Cox regression model with treatment status as a time-varying covariate.

A total of 6548 sero-discordant couples were included in the cohort study analysis. Among them, 348 negative partners sero-converted. HIV seroincidence was significantly higher among the nontreated (4.3 per 100 person-years, 3.7–4.9) compared with those receiving ART (1.8 per 100 person-years, 1.5–2.0). An overall 35% reduction in risk of HIV transmission was associated with receiving ART (adjusted hazard ratio [AHR] 0.65, 95% confidence interval [CI] 0.51–0.83), and the yearly risk reduction was also significant in the first 3 consecutive years of follow-up. Moreover, ART was found to be significantly inversely associated with multiple baseline characteristics of index partners.

TasP may be feasible on a national or regional scale. In addition to other proven preventive strategies such as the use of condoms, ART adherence to maintain viral suppression would then be the key challenge for successful TasP implementation.

## INTRODUCTION

Antiretroviral therapy (ART) has significantly improved the prognosis of patients infected with human immunodeficiency virus type 1 (HIV-1) and decreased associated morbidity and mortality.^1–6^ ART also can reduce transmission of HIV-1 among HIV serodiscordant couples.^7–11^ The HIV Prevention Trials Network 052 (HPTN 052) study definitively showed the benefit of ART for the prevention of sexual transmission of HIV in serodiscordant heterosexual couples in 2011.^12^ Since the publication of this trial result, the World Health Organization (WHO) has recommended ART as a method for preventing HIV transmission among serodiscordant couples.^13^ Recently, a Chinese national observational cohort study showed risk reduction in HIV transmission among serodiscordant couples in a real-world setting, suggesting that the so-called treatment-as-prevention (TasP) approach is a possible and effective public health HIV prevention strategy.^14^

Unlike the HPTN 052 being a well-designed controlled randomized trial for a certain eligible population, key research challenges remain to assess the TasP effectiveness in real-world settings. In the aforementioned Chinese national observational cohort study, for example, TasP effectiveness was assessed by only comparing the follow-up time of index partners who never received any ART with the follow-up time of index partners after they had initiated ART; thus, the follow-up time of index patients from the baseline visit to their initiation of ART was not included for analysis. (“Index partner” refers to individuals who were HIV-positive at baseline, but whose partner was HIV-negative). Such an assessment might result in a bias towards couples who are inherently less likely to transmit. To address this issue of possible bias in real-world settings, we use an extended time-dependent Cox regression model to conduct a cohort study analysis of serodiscordant couples in Guangxi of China. In this model, whether the index partner receives ART or not will be treated as a time-dependent covariate in the extended Cox model. The aim of this study is thus to more accurately assess TasP effectiveness on the transmission of HIV among serodiscordant couples in a real-world setting, such as Guangxi, China.

The HIV epidemic among drug users was first detected in 1996 in Guangxi. By the end of 2004, Guangxi had accumulated the third highest number of HIV cases reported in China.^21^ HIV infection through drug injection accounted for 69% of reported HIV cases in 2003, but the proportion of HIV infections through sexual intercourse increased to 66% in 2009 in Guangxi.^22,23^ Since the Chinese National Comprehensive AIDS (acquired immune deficiency syndrome) Response Policy and “Four Frees and One Care” program were launched in 2003,^24^ the Chinese Center for Disease Control and Prevention (China CDC) has established national HIV databases. These cohort study databases can be used to study the effectiveness of ART on the transmission of HIV in serodiscordant couples.

## MATERIALS AND METHODS

### The Sampling Method

**FIGURE 1:**
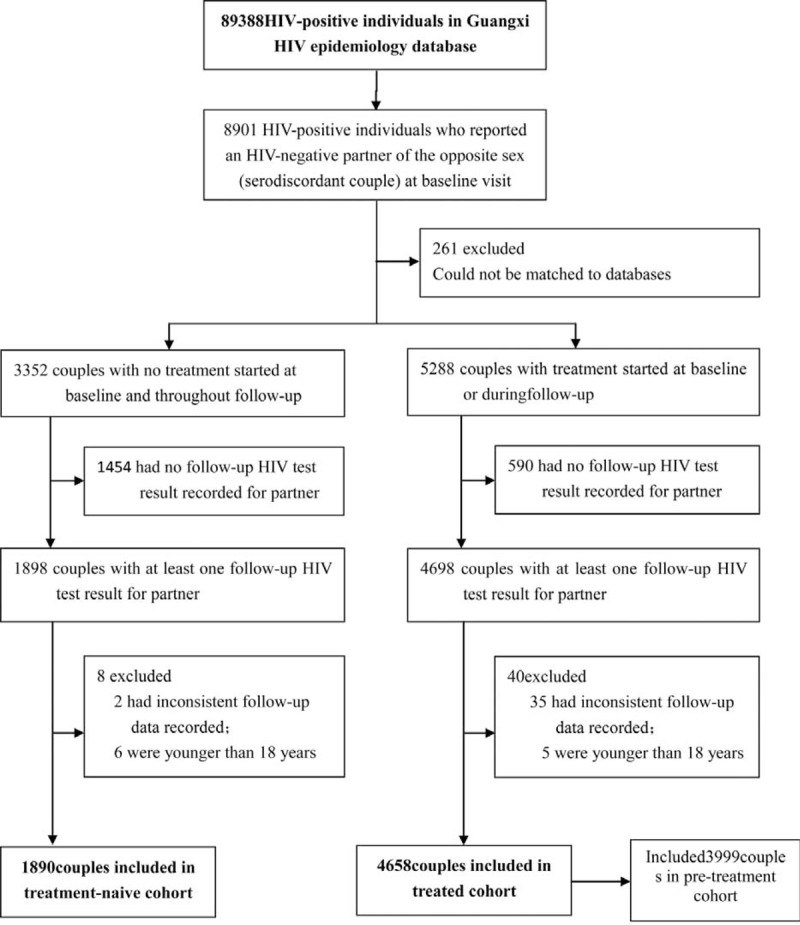
Study profile.

This observational cohort study of HIV serodiscordant couples was conducted primarily in rural regions of Guangxi province, Southwest China, and used data collected from 2003 to 2013. HIV-positive individuals were followed up every 6 months for repeated CD4 counts and clinical evaluation. The local CDC followed up individuals who had a spouse or regular sex partner, and tested them for HIV every 6 months for partners who tested negative. The national treatment criterion in 2008 was CD4 cell count < 350/mm^3^ (cutoff increased from 200 cells per μL in 2008); WHO stage III/IV diseases; or willingness to receive ART, regardless of the criteria 1 and 2. Serodiscordant couples were defined by two criteria: at baseline, 1 individual was HIV-positive (index patient), while their partner tested HIV-negative; and at the partner who tested HIV-negative at baseline had at least 1 additional follow-up HIV test result. In order to assess seroconversion rates overtime among HIV serodiscordant couples, we retrospectively analyzed national HIV epidemiology and treatment databases. Individuals in the treatment database were linked to their own records in the epidemiology database by the use of their unique treatment identification numbers or national identification numbers. Data linkage between the epidemiology and treatment databases was verified twice in a relational database. Additional details concerning these cohort study databases have been previously published elsewhere.^2,3,25^ By the end of 2013, 89,388 HIV-positive individuals were reported to the Guangxi HIV database. After exclusion of couples who did not meet the study criteria, 1890 couples were included in the treatment-naive cohort, and 4658 couples were included in the treated cohort (Figure 1).

### Data Collection

Study participants in the HIV treatment cohort study database were linked to their own records in the HIV epidemiology database using their unique national identification numbers or treatment identification numbers. According to their treatment status, the serodiscordant couples of study participant could be classified into 3 groups: those never treated (group 1), those who entered untreated but subsequently began treatment during follow-up (group 2), and those treated immediately upon entry into the public health system (group 3). Baseline characteristics of all index patients were extracted from the databases, such characteristics included sex, age, education status, marital status, occupation, and route of transmission. CD4 count at the baseline visit was extracted for those never treated in group 1 and before treatment in group 2, respectively. CD4 count at the visit before initializing treatment was extracted for those after treatment in group 2 and in group 3.

### Statistics Method

The cohort study endpoint was time to incidental HIV seroconversion. Kaplan-Meier analysis was used to calculate the HIV-infection-free survival probabilities for the HIV-negative partners. In order to assess treatment effectiveness, we estimated the treatment hazard ratios (HRs; reported with 95% confidence intervals [CIs]) by using univariate and multivariate extended Cox regression models with the time-varying covariate of treatment status. In order to control for bias, the following factors were included in the adjusted models: duration of follow-up, sex, age, education, marital status, occupation, route of HIV infection, and baseline CD4 cell count of the index patient. A 2-sided *P*-value of 0.05 or less was regarded as statistically significant. The data were analyzed using Statistical Analysis System (SAS 9.1 for Windows; SAS Institute Inc., NC).

### Ethics Statement

The study was approved by the institutional review board of the Guangxi Center for Disease Control and Prevention.

## RESULTS

**FIGURE 2:**
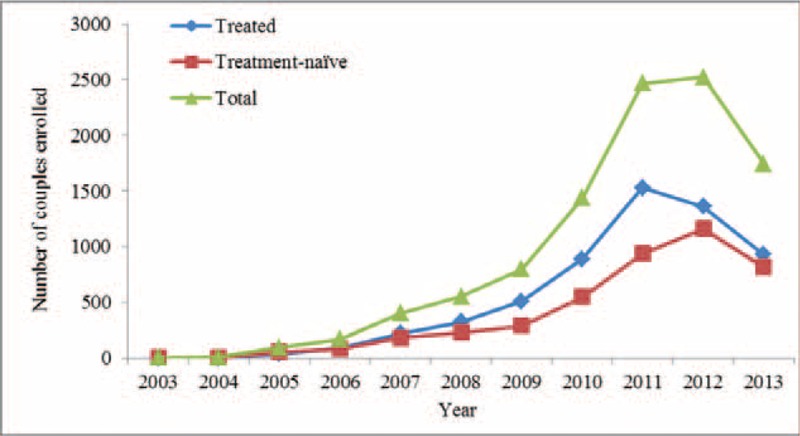
Serodiscordant couples enrolled, by year.

A total of 6548 couples were included in the cohort study analysis. The numbers of participants were 6200 for nonseroconversion couples and 348 for seroconversion couples, respectively. The number of serodiscordant couples enrolled by year is illustrated in Figure 2. Table 1 presents the baseline characteristics of serodiscordant couples who did and did not experience a seroconversion during the study observation period. The proportion of males was 74% in the nonseroconversion group and 64% in the seroconversion group. Age above 45 years represented 38% of the nonseroconversion group and 36% of the seroconversion group. Fourteen percent of those with postsecondary education were in the nonseroconversion group and 12% were in the seroconversion group. Ninety three percent of the nonseroconversion group was married compared with 94% in those of seroconversion group. The proportion of farmers was 62% in the nonseroconversion group and 64% in the seroconversion group in the proportion of index patients infected through heterosexual intercourse was 89% and 87% in the nonseroconversion group and seroconversion group, respectively. Significant differences between the nonseroconversion group and seroconversion group were found for sex and CD4 cell count in the index patients (*P* < 0.05).

**TABLE 1:**
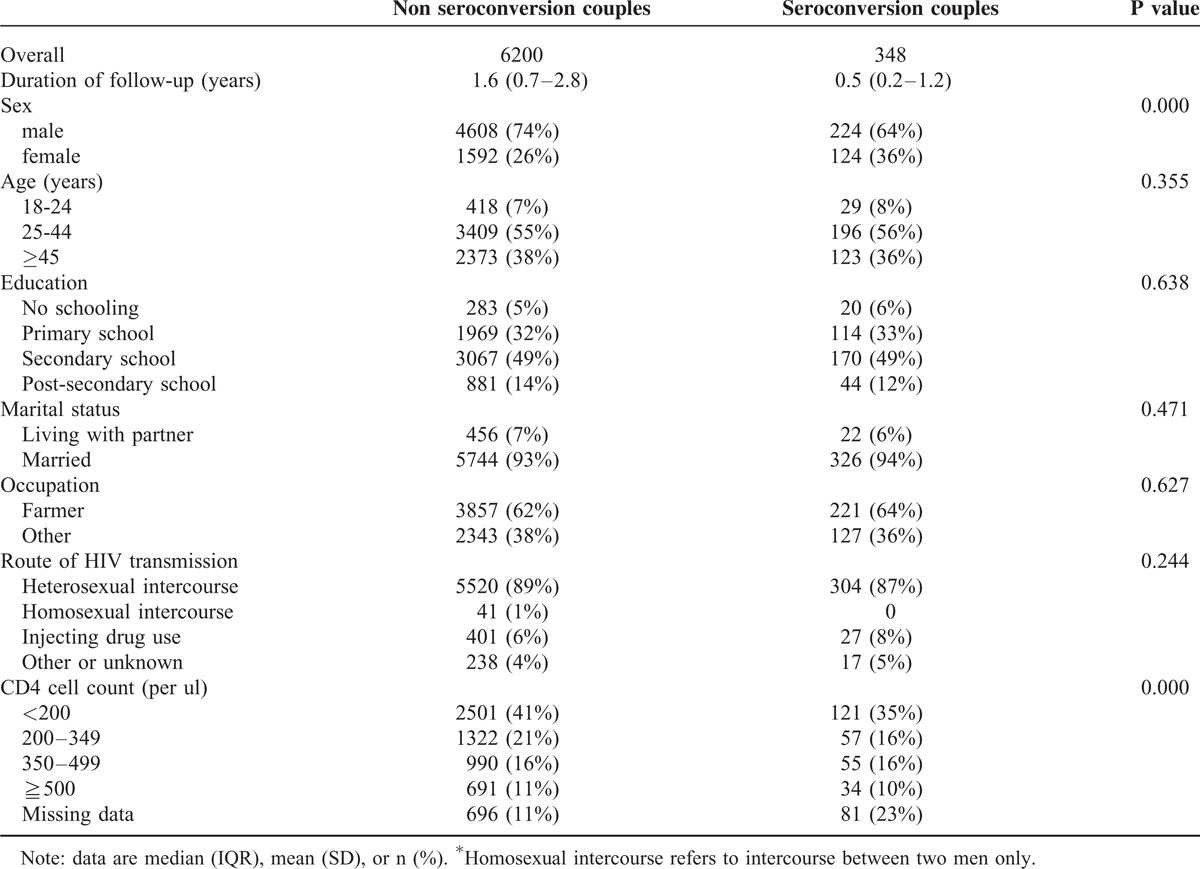
Study participant sociodemographics by seroconversion status

**FIGURE 3:**
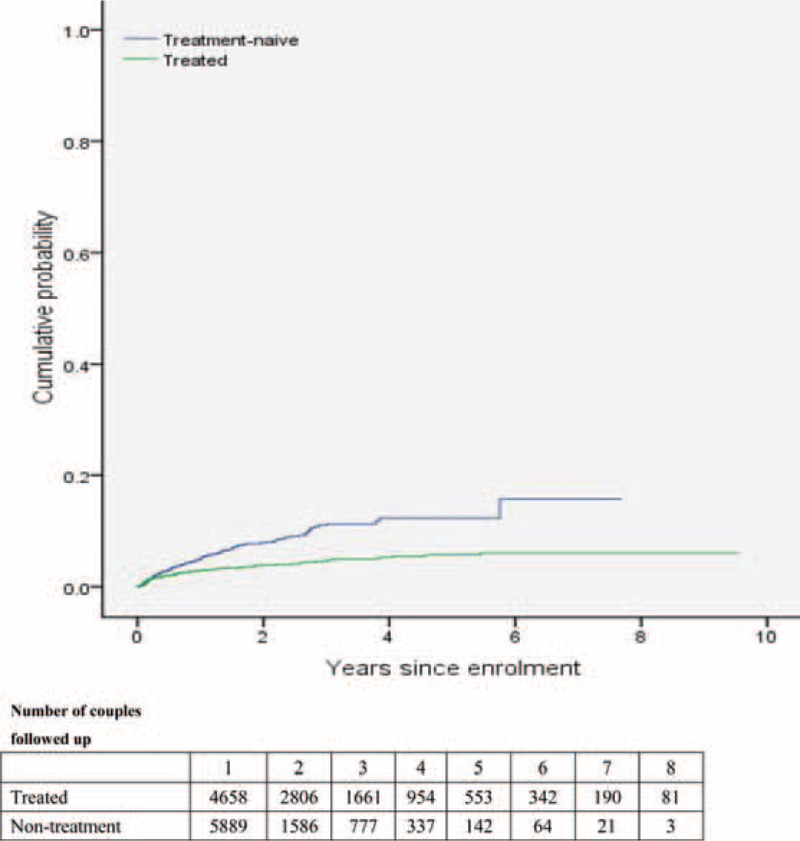
Cumulative probability of HIV-negative partner in serodiscordant couple becoming infected with HIV, by number of years since enrolment.

By the end of 2013, a total of 348 HIV seroconversions were observed during the follow-up period. Table 2 shows that the average incidence of HIV was 2.6 per 100 person-years (95% CI 2.4–2.9). HIV seroincidence was significantly higher among the nontreatment group (4.3 per 100 person-years, 3.7–4.9) compared with the treatment group (1.8 per 100 person-years, 1.5–2.0). An overall 35% reduction in the risk of HIV transmission was associated with antiretroviral treatment (adjusted hazard ratio [AHR] 0.65, 95% CI 0.51–0.83; Table 3). The difference in the cumulative probability of seroconversion for the HIV-negative partner increased overtime between the nonseroconversion and seroconversion groups (Figure 3).

**TABLE 2:**
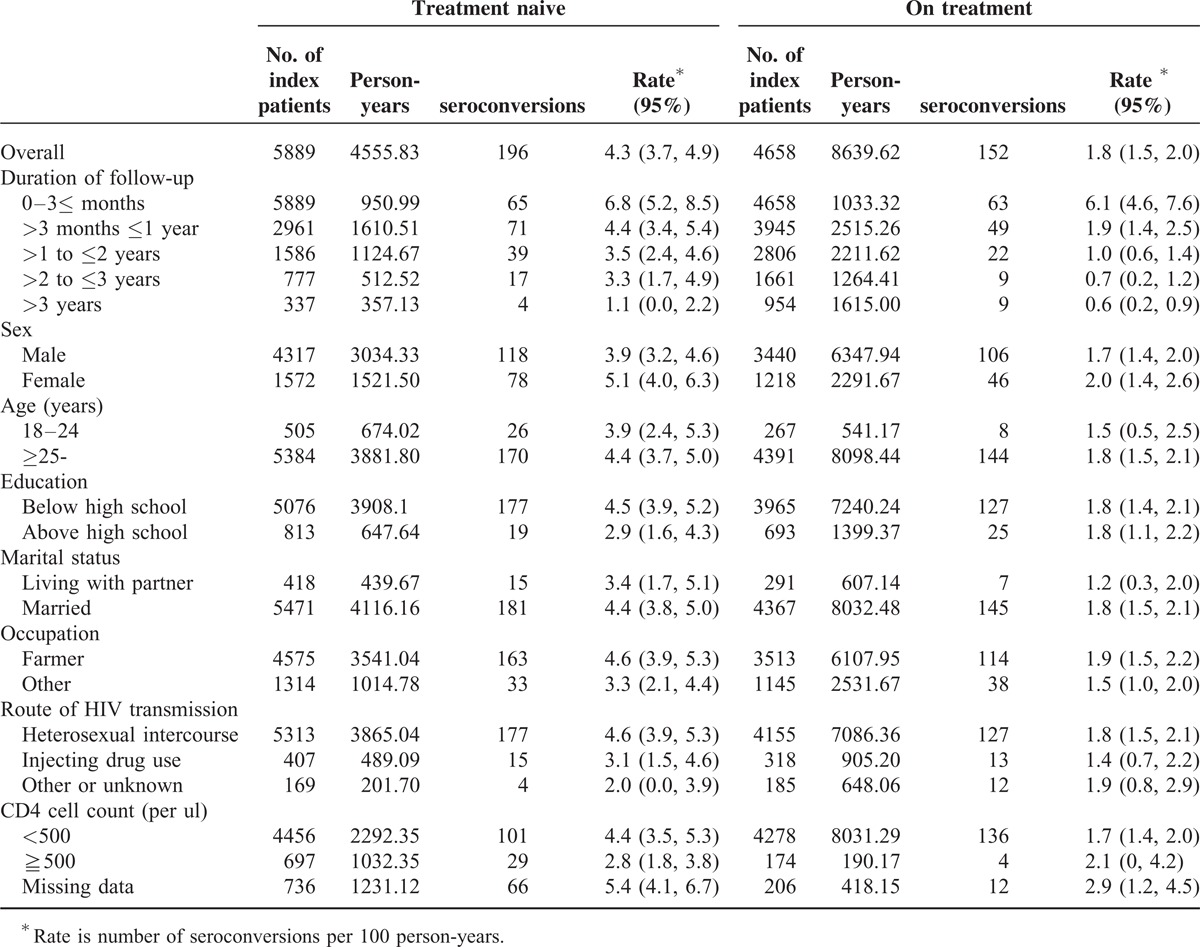
Comparison of seroconversion rates by treatment history, duration of follow-up, sociodemographics, and CD4 count

**TABLE 3:**
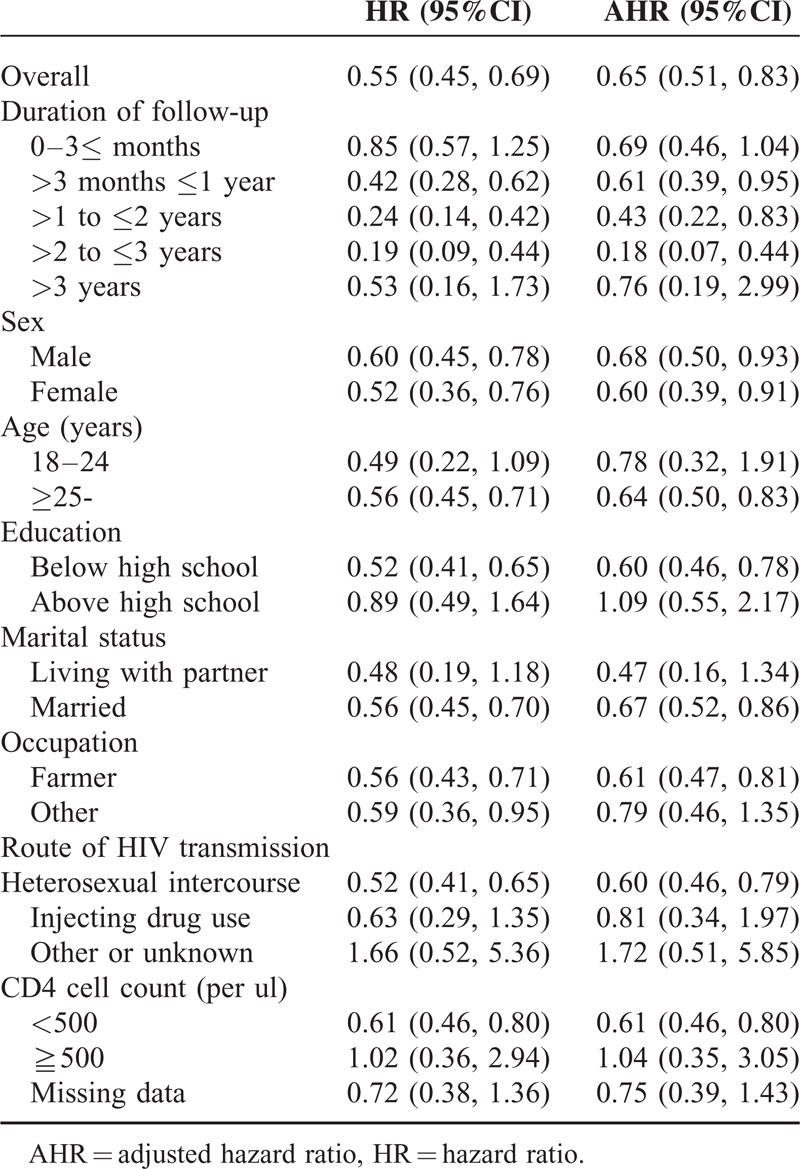
Comparison of Cox regression model analyses stratified by duration of follow-up and baseline characteristics of the index patient (HIV-positive partner)

Due to some differences of baseline characteristics between the nontreatment and treatment groups in real-world setting, we estimated rates of HIV incidence stratified by the baseline variables for subgroup analysis, and adjusted the HRs to control for potential confounding (Tables 2 and 3). The results of the adjusted Cox regression model showed that HIV incidence stratified by baseline characteristics was most significantly protective for the treatment group. Treatment was significantly preventive in the 1st (>3 months ≤1 year), 2nd and 3rd year of follow-up (AHR 0.61, 95% CI 0.39–0.95; AHR 0.43, 95% CI 0.22–0.83; AHR 0.18, 95% CI 0.07–0.44), but not in the 4th year (AHR 0.76, 95% CI 0.19–2.99) or in subsequent years.

Treatment was found to be significantly inversely associated with several baseline characteristics of index partners: male sex (0.68, 0.50–0.93), female sex (0.60, 0.39–0.91), age above 25 years (0.64, 0.50–0.83), less than high school education (0.60, 0.46–0.78), being married (0.67, 0.52–0.86), being a farmer (0.61, 0.47–0.81), where the index partner had been infected by heterosexual intercourse (0.60, 0.46–0.79), and baseline CD4 counts of 500 cells per μL or less (0.61, 0.46–0.80). No significant differences between nontreatment and treatment groups were found for the following baseline characteristics of index partners: age between 18 and 24 years, education above high school, living with a partner, not being a farmer, infected by injection drug use or other means, and CD4 counts of 500 cells per μL and above Table 3.

## DISCUSSION

Our study found a 35% reduction in HIV transmission in couples where the index patients had received ART. A small cohort study in rural Yunnan province of China showed that serodiscordant partners of HIV-positive patients receiving ART had about one third of the risk of HIV transmission compared to those of HIV-positive patients not receiving ART.^26^ Another recent cohort study with the HIV-positive index patients, mainly former plasma donors in Henan province of China found ART was highly protective against seroconversion when the former plasma donor index patient was actively receiving ART treatment at the last follow-up (adjusted rate ratio [RR] = 0.05, 95% CI 0.01–0.16).^27^ However, this study had a large imbalance between patients with treatment and no treatment, due to RR = 0.36 (95% CI, 0.24, 0.55) in univariate Cox regress analysis. The previous study results showed a 26% relative reduction in HIV in the treated cohort of Chinese national serodiscordant couples (2003–2011).^14^ Based on the results of HPTN 052, our study strongly supports the public health prevention strategy of TasP among serodiscordant couples under real-world conditions on a national or regional scale.^13^

Our study results showed that treatment was significantly protective in the first 3 years of follow-up and was protective across most baseline socio-demographic characteristics, with a few exceptions. This finding updated our previous study that protection was only significant in the first year. Exactly how durable this protection is remains unclear. Some factors associated with duration of this protection should be considered in future studies: future cohort studies need statistical power to measure this long-term protection overtime, including sufficient numbers of seroconversions over the observational period. This reduction in risk could also be related to the natural history of HIV transmission among serodiscordant couples, as those susceptible to infection usually decreases overtime.^28,29^ Other reason is that drug resistance to ART increased overtime.^30–33^ In order to guide public health decision making, future studies are urgently needed to clarify the duration of protection conferred by TasP.

Another major finding of our study was that treatment reduced HIV transmission when the index partners’ CD4 cell counts were below 500 before treatment. TasP may be less beneficial to those whose index partners had relatively high CD4 cell counts before initializing ART. The impact of higher CD4 cell count on virologic failure warrants further investigation. The Chinese treatment guideline has changed and advocates initiating ART treatment at higher CD4 levels (above 350), if patients are willing to receive ART and will have good adherence.^34^ A few international studies showed that early treatment led to virological and clinical benefits.^35,36^ However, 1 national cohort study in China has also shown that patients with higher CD4 cell counts at ART initiation were more likely to drop out of HIV care,^37^ and another prospective follow-up study in China found that patients with higher CD4 counts before treatment were at increased risk for poor virological response.^38^ Additional studies are needed to clarify the effects of early ART initiation on adherence and attrition at short-term versus long-term treatment timescales. Such studies could help to address the issues of initiating ART treatment at high CD4 cell counts.

Our study validated reduction in the risk of HIV transmission in couples wherein index patients received ART. Treatment was significantly protective for most baseline characteristics of index partners, such as for male, female, age above 25 years, education below high school, married, farmer, and infected by heterosexual intercourse. Successful implementation of a strategy for HIV TasP will depend on its ability to be implemented in real-world scenarios. It is an urgent need to provide comprehensive harm reduction measures to serodiscordant couples such as condoms, methadone maintenance therapy (MMT), needle exchanges, and STD management services.^39^ Such programs help reduce HIV and sexual transmitted diseases among high risk groups of injection drug uses and female sex workers in China.^40,41^ ART adherence, education, and interventions are a key challenge for successful implementing TasP strategies in real-world settings.

Our study has several limitations. First, although we controlled for numerous confounders in multivariate modeling, differences in baseline characteristics among the 3 groups suggest that selection bias may have led to overestimation of the ART's true impact. Second, the median duration of follow-up is relatively short, and the long-term durability of TasP needs to be clarified in future studies. Third, some variables in our study are self-reported, so some misreporting or underreporting may have happened. Our study further strengthens support for ART's preventive effect in the real-world, but ART adherence and risk behavior reduction while implementing TasP require further study.

### Uncited references

^15–20^

## References

[1] MacArthur RD Novak RM Peng G A comparison of three highly active antiretroviral treatment strategies consisting of non-nucleoside reverse transcriptase inhibitors, protease inhibitors, or both in the presence of nucleoside reverse transcriptase inhibitors as initial therapy (CPCRA 058 FIRST Study): a long-term randomised trial. *Lancet* 2006; 368:2125–2135. 17174704 10.1016/S0140-6736(06)69861-9

[2] Zhang F Dou Z Ma Y Effect of earlier initiation of antiretroviral treatment and increased treatment coverage on HIV-related mortality in China: a national observational cohort study. *Lancet Infect Dis* 2011; 11:516–524. 21600849 10.1016/S1473-3099(11)70097-4

[3] Zhang F Dou Z Yu L The effect of highly active antiretroviral therapy on mortality among HIV-infected former plasma donors in China. *Clin Infect Dis* 2008; 47:825–833. 18690805 10.1086/590945 PMC2538607

[4] Liao L Xing H Su B Impact of HIV drug resistance on virologic and immunologic failure and mortality in a cohort of patients on antiretroviral therapy in China. *AIDS* 2013; 27:1815–1824. 23803794 10.1097/QAD.0b013e3283611931 PMC3694318

[5] Zhang F Dou Z Ma Y Five-year outcomes of the China National Free Antiretroviral Treatment Program. *Ann Intern Med* 2009; 151:241–251.W-52. 19687491 10.7326/0003-4819-151-4-200908180-00006

[6] Zhu H Napravnik S Eron JJ Decreasing excess mortality of HIV-infected patients initiating antiretroviral therapy: comparison with mortality in general population in China, 2003–2009. *J Acquir Immune Defic Syndr* 2013; 63:e150–e157. 23572014 10.1097/QAI.0b013e3182948d82 PMC3752313

[7] He N Duan S Ding Y Antiretroviral therapy reduces HIV transmission in discordant couples in Rural Yunnan, China. *PLoS One* 2013; 8:e77981. 24236010 10.1371/journal.pone.0077981 PMC3827220

[8] Wang L Peng ZH Li LM HIV seroconversion and prevalence rates in heterosexual discordant couples in China: a systematic review and meta-analysis. *AIDS Care* 2012; 24:1059–1070. 22452488 10.1080/09540121.2012.661837

[9] Fang CT Hsu HM Twu SJ Decreased HIV transmission after a policy of providing free access to highly active antiretroviral therapy in Taiwan. *J Infect Dis* 2004; 190:879–885. 15295691 10.1086/422601

[10] Quinn TC Wawer MJ Sewankambo N For the Rakai Project Study Group. Viral load and heterosexual transmission of human immunodefi ciency virus type 1. *N Engl J Med* 2000; 342:921–929. 10738050 10.1056/NEJM200003303421303

[11] Fideli US Allen SA Musonda R Virologic and immunologic determinants of heterosexual transmission of human immunodefi ciency virus type 1 in Africa. *AIDS Res Hum Retroviruses* 2001; 17:901–910. 11461676 10.1089/088922201750290023 PMC2748905

[12] Cohen MS Chen YQ McCauley M Prevention of HIV-1 infection with early antiretroviral therapy. *N Engl J Med* 2011; 365:493–505. 21767103 10.1056/NEJMoa1105243 PMC3200068

[13] WHO. Guidance on couples HIV testing and counselling including antiretroviral therapy for treatment and prevention in serodiscordant couples: recommendations for a public health approach. Geneva: World Health Organization, 2012 http://apps.who.int/iris/bitstream/10665/44646/1/9789241501972_eng.pdf (accessed June 7, 2012). 23700649

[14] Jia Z Mao Y Zhang F Antiretroviral therapy to prevent HIV transmission in serodiscordant couples in China (2003-11): a national observational cohort study. *Lancet* 2013; 382:1195–1203. 23206835 10.1016/S0140-6736(12)61898-4

[15] Wei L Chen J Rodolph M HIV incidence, retention, and changes of high-risk behaviors among rural injection drug users in Guangxi, China. *Subst Abus* 2006; 27:53–61. 10.1300/j465v27n04_07 17347126

[16] Lai S Liu W Chen J Changes in HIV-1 incidence in heroin users in Guangxi Province, China. *J Acquir Immune Defic Syndr* 2001; 26:365–370.PubMed PMID: 11317080. 11317080 10.1097/00126334-200104010-00014

[17] Lai S Chen J Celentano D Adoption of injection practices in heroin users in Guangxi Province, China. *J Psychoactive Drugs* 2000; 32:285–292.PubMed PMID: 11061679. 11061679 10.1080/02791072.2000.10400451

[18] Yu XF Chen J Shao Y Emerging HIV infections with distinct subtypes of HIV-1 infection among injection drug users from geographically separate locations in Guangxi Province, China. *J Acquir Immune Defic Syndr* 1999; 22:180-188 PubMed PMID: 10843533. 10843533 10.1097/00126334-199910010-00011

[19] Yu XF Chen J Shao Y Two subtypes of HIV-1 among injection-drug users in southern China. *Lancet* 1998; 351:1250 PubMed PMID: 9643749. 9643749 10.1016/S0140-6736(05)79316-8

[20] Beyrer C Razak MH Lisam K Overland heroin trafficking routes and HIV-1 spread in south and south-east Asia. *AIDS* 2000; 14:75–83. 10714570 10.1097/00002030-200001070-00009

[21] Guangxi Public Health Department. Annual Report on Provincial AIDS/STD in 2004. Guangxi: Guangxi Public Health Department; 2005.

[22] Guangxi Public Health Department. Annual Report on Provincial AIDS/STD Surveillance in 2003. Guangxi: Guangxi Public Health Department; 2004.

[23] Guangxi Public Health Department. Annual Report on Provincial AIDS/STD in 2009. Guangxi: Guangxi Public Health Department; 2010.

[24] Han MG Chen QF Han Y Design and implementation of a China comprehensive AIDS response programme (China CARES), 2003-08. *International Journal of Epidemiology* 2010; 39:ii47–ii55. 21113037 10.1093/ije/dyq212 PMC2992617

[25] Ma Y Zhang F Zhao Y Cohort profi le: the Chinese national free antiretroviral treatment cohort. *Int J Epidemiol* 2010; 39:973–979. 19556327 10.1093/ije/dyp233 PMC2929349

[26] He N Duan S Ding Y Antiretroviral Therapy Reduces HIV Transmission in Discordant Couples in Rural Yunnan, China. *PLoS One* 2013; 8:e77981 doi: 10.1371/journal.pone.0077981. 24236010 10.1371/journal.pone.0077981 PMC3827220

[27] Wang L Wang L Smith MK Heterosexual transmission of HIV and related risk factors among serodiscordant couples in Henan province, China. *Chin Med J (Engl)* 2013; 126:3694–3700.PubMed PMID: 24112166. 24112166

[28] Collaborative Group on, AIDS., Incubation and, HIV., Survival, including the, CASCADE., EU., Concerted, Action., Time from HIV-1 seroconversion to, AIDS., and death before widespread use of highly-active antiretroviral therapy: a collaborative, re-analysis. *Lancet* 2000; 355:1131–1137. 10791375

[29] Wawer MJ Gray RH Sewankambo NK Rates of HIV-1 transmission per coital act, by stage of HIV-1 infection, in Rakai, Uganda. *J Infect Dis* 2005; 191:1403–1409. 15809897 10.1086/429411

[30] Xing H Ruan Y Li J HIV drug resistance and its impact on antiretroviral therapy in Chinese HIV-infected patients. *PLoS One* 2013; 8:e54917 doi: 10.1371/journal.pone.0054917. 23405098 10.1371/journal.pone.0054917 PMC3566114

[31] Xing H Wang X Liao L Incidence and associated factors of HIV drug resistance in Chinese HIV-infected patients receiving antiretroviral treatment. *PLoS One* 2013; 8:e62408 doi: 10.1371/journal.pone.0062408. 23638072 10.1371/journal.pone.0062408 PMC3640055

[32] Liao L Xing H Shang H The prevalence of transmitted antiretroviral drug resistance in treatment-naive HIV-infected individuals in China. *J Acquir Immune Defic Syndr* 2010; 53 suppl 1:S10–14. 20104099 10.1097/QAI.0b013e3181c7d363 PMC3422681

[33] Liao L Xing H Su B Impact of HIV drug resistance on virologic and immunologic failure and mortality in a cohort of patients on antiretroviral therapy in China. *AIDS* 2013; 27:1815–1824. 23803794 10.1097/QAD.0b013e3283611931 PMC3694318

[34] Manual of the National Free Antiretroviral Treatment, third edition. Available at: http://www.chinaaids.cn/jszn/201301/t20130111_75763.htm

[35] Uy J Armon C Buchacz K HOPS Investigators. Initiation of HAART at higher CD4 cell counts is associated with a lower frequency of antiretroviral drug resistance mutations at virologic failure. *J Acquir Immune DeficSyndr* 2009; 51:450–453. 10.1097/QAI.0b013e3181acb630 19474757

[36] Harrigan, PRUK., Collaborative Group on, HIV., Drug Resistance;, UKCHIC., Study, Group., Long-term probability of detecting drug-resistant, HIV., in treatment-naive patients initiating combination antiretroviral, therapy. *Clin Infect Dis* 2010; 50:1286–1287.10.1086/651685. 20353366 10.1086/651684

[37] Zhu H Napravnik S Eron J Attrition among human immunodeficiency virus (HIV)- infected patients initiating antiretroviral therapy in China. *PLoS One* 2012; 7:e39414 doi: 10.1371/journal.pone. 22761787 10.1371/journal.pone.0039414 PMC3384674

[38] Wang J He C Hsi JH Virological outcomes and drug resistance in Chinese patients after 12 months of 3TC-based first-line antiretroviral treatment. *PLoS One* 2014; 9:e88305 doi: 10.1371/journal.pone.0088305. 24516631 10.1371/journal.pone.0088305 PMC3917868

[39] Han MG Chen QF Han Y Design and implementation of a China comprehensive AIDS response programme (China CARES). *International Journal of Epidemiology* 2010; 39:ii47–ii55. 21113037 10.1093/ije/dyq212 PMC2992617

[40] Ruan Y Liang S Zhu J Evaluation of harm reduction programs on seroincidence of HIV, hepatitis B and C, and syphilis among intravenous drug users in southwest China. *Sex Transm Dis* 2013; 40:323–328. 23486498 10.1097/OLQ.0b013e31827fd4d4

[41] Zhang L Liang S Lu W HIV, syphilis, and behavioral risk factors among female sex workers before and after implementation of harm reduction programs in a high drug-using area of China. *PLoS One* 2014; 9:e84950 doi: 10.1371/journal. 24416319 10.1371/journal.pone.0084950 PMC3885653

